# Event and Comment

**Published:** 1910-06-15

**Authors:** 


### DENTAL REGISTER.

**Vol. LXIV. June 15, 1910. No. 6.**

### EVENT AND COMMENT.

---

**PROFESSIONAL EDUCATION.** There never has been a time when in medicine and dentistry there was so strong a sentiment and tendency in the profession and on the part of the managers of our educational affairs to exact higher educational standards. There are many reasons for this which we can only take the space to mention. There is in the first place a general toning up of standards in all educational institutions which are established on other than commercial bases. High school standards of today are more rigidly kept up to a high grade because of the demands and inclinations of the people for higher ideals, but more especially because the high school is rapidly becoming the standard preparation for admission to colleges and universities; and by state laws students can not be admitted to medical and dental colleges without high school education. In the second place there is no longer a dearth, especially of medical practitioners and there is no good reason why low standards should prevail. Members of the profession are at last realizing that both medicine and dentistry of today require a broader culture than ever before, because of the continual expansion of both the scientific and technical departments. Some have thought the strong sentiment in favor of higher educational requirements may be due to the strong competition of crowded professions, and that it has no high basis, but rather arises from the selfish motive of self preservation. But when one finds this sentiment strongest in the comments of those who have no such selfish interest to bias their judgements, such as scientists, and teachers, we must conclude that it is a real need and that it has a worthy conception.

We are printing in this issue two articles by eminent educators, whose motives no one will question, as to the needs, as these great and experienced educators see them.

The articles were read before the Council on Medical Education of the American Medical Association and while they are of considerable length and treat only of Medical Education, they so aptly meet our Dental Educational needs as to make them well worth our serious consideration. Necessarily some will question the advisability of putting our dental standard on the same basis as our older and more advantaged medical institutions. We find that these two educators are not ready to say for instance that medical schools should all require an academic degree for admission and four years of medical science and technical training. And yet Dr. Schurman intimates that the academic degree and a four year medical course with one year of post-graduate hospital practice would be ideal for the making of a country doctor; but that it might be the means of encroaching unduly on the students time. We are not so certain that it is absolutely essential that a young man should get his preparation for his life work made before he has reached an age of discretion, say from 25 to 30 years. Most young men are glad to get fairly well started in business at thirty, and we believe any one with a broad or liberal education even in a technical profession will accomplish more, before he is compelled to retire because of physical infirmities. There can be little doubt but a well balanced and selected course of classical and scientific training before taking up a course in dentistry would be tremendously advantageous. However it is not always the man with the academic degree that accomplishes most. The late Professor W. D. Miller, with all his humanitarian interest and professional passion could never have accomplished his wonderful work except for the fine academic training he obtained before taking up dentistry. Natural and inherent ability and the power of self-denial count sometimes for as much as academic training as we can see well illustrated in the lives of many of our most illustrious men, more perhaps in Prof. G. V. Black than any other man in our profession. There can, however, be little question but that for the average men, it will be much safer for us to predict greater attainments from our profession in the future, if we can better prepare the men who are to have its making in their care. The practical question of course is to procure educated men as students and then to have educated faculties with proper facilities for making clear to them the work which they are to undertake. Can we jump at once to graduation from colleges in place of high school graduation as an entrance requirement? We could do so undoubtedly if the profession was united in its judgement and would support the schools with proper endowments and instructors. In our experience we have found the profession practically of one mind as to higher entrance and graduate requirements, but sadly indifferent when asked to provide the means for carrying out such advancement. No school has had sufficient patronage in endowment or even well prepared instructors to handle this proposition on the high standard that all seem agreed it should be maintained. It's clearly a case of where both "money and brains talk". No great amount of endowment for dental education is in sight at present, and no considerable number of young men seem to be sacrificing their financial opportunities to prepare themselves to instruct the future dentists. Can we as a profession expect to shift the burden of this work to the literary and medical science faculties and expect to secure graduates who shall measure up to our ideals? It seems to us that we must take short steps but such as shall in time lead to our goal.

